# The Phenomenon of “Obesity Paradox” in Neck of Femur Fractures

**DOI:** 10.12669/pjms.36.5.1952

**Published:** 2020

**Authors:** Muhammad Tahir, Nadeem Ahmed, Muhammad Qasim Ali Samejo, Allah Rakhio Jamali

**Affiliations:** 1 Mr. Muhammad Tahir, MRCS Eng. Department of Orthopaedics, Surgical Building, Jinnah Postgraduate Medical Centre, Rafiqui Shaheed Road, Karachi, Pakistan; 2 Dr. Nadeem Ahmed, FCPS. Department of Orthopaedics, Surgical Building, Jinnah Postgraduate Medical Centre, Rafiqui Shaheed Road, Karachi, Pakistan; 3 Dr. Muhammad Qasim Ali Samejo, FCPS. Department of Orthopaedics, Surgical Building, Jinnah Postgraduate Medical Centre, Rafiqui Shaheed Road, Karachi, Pakistan; 4 Prof. Allah Rakhio Jamali Department of Orthopaedics, Surgical Building, Jinnah Postgraduate Medical Centre, Rafiqui Shaheed Road, Karachi, Pakistan

**Keywords:** Hip fractures, Mortality, Obesity paradox

## Abstract

**Objective:**

To determine the association of body mass index (BMI) with 30 days and 1-year mortality outcomes of orthopedic elderly patients after hip fracture surgery.

**Methods:**

This is prospective study conducted at Department of Orthopaedics, at a tertiary care public sector hospital in Karachi between Jan-2016 to Jan-2018. In this short follow-up study, we included the data of 490 patients, who were operated for neck of femur fractures in a public sector tertiary care hospital between Jan-2016 to Jan-2018. Patients were divided into different categories on the basis of BMI; BMI <20 Kg.m^-2^ underweight, 20-24.99 Kg.m^-2^ normal weight, BMI 25-29.99 Kg.m^-2^ overweight, ≥30 obese. Mortality at 30 days and 1-year mortality were primary study end-points.

**Results:**

Rate of re-admission within 30 days, major adverse cardiovascular events (MACE) within 30 days and 30 days mortality was high in underweight and lowest in obese patients. Thirty-day mortality rate was 2.7% in underweight, 1.3% in normal weight, 0.64% in over-weight and 0.0% in obese patients but this was not significant statistically (p-value 0.29). One-year mortality rate was significantly high in under-weight patients, 34.2%, 25.9% in normal weight, 21.4% in overweight and only 14.5% in obese patients (p-value 0.009). Age ≥ 65 years (odds ratio 0.40 (0.26-0.63), and ASA III-IV (odds ratio; 0.27 (0.16-0.45) are also significant risk factors of 1-year mortality

**Conclusion:**

BMI classification can serve as an important indicator of adverse early outcomes after hip fracture surgery. Over-weight and obese patients have better survival outcomes and have lower 1-year mortality rate.

## INTRODUCTION

There has been an increasing prevalence of obesity in the last 25 years.^1^ The prevalence increased to 38% in 2014 from 12% in U.S,^2^,^3^ and 27% in 2015 from 15% in U.K.^4^ Obesity has several adverse effects on human health such as increased risk of ischemic heart disease, diabetes mellitus, hypertension and stroke and all-cause mortality in normal population.^5^-^8^ Average body mass index (BMI) is increasing all around the world, and this global health problem is now about to achieve the level of an epidemic.

Some studies have reported better outcomes in over-weight and obese patients of older age.^9^,^10^ This phenomenon is termed as “obesity paradox.^11^ The over-all incidence of death has been shown to decline with an advancing age.^12^ In older age, risk of mortality is significantly higher in under-weight patients as compared to the over-weight and obese patients.^13^,^14^

The underlying mechanism behind obesity paradox is still unclear. It may be because being overweight or obese indicate the absence of chronic diseases that may not be so much evident in young age patients. Hip fractures initiate a series of stress response and metabolic disturbances for a prolonged time period.^15^ Presence of surplus adipose tissues in obese patients serve as metabolic reserves during such hostile events. Patients with higher BMI may therefore be more tolerant to stressful events such as trauma or any other critical illness.^16^

A large number of elderly patients are treated in every orthopedic department, and the proportion of elderly patients coming to the orthopedic departments for hip fractures surgeries has increased considerably in last two decades in Pakistan.^17^ Despite this obesity paradox is still not widely explored in this specialty.

The aim of present study was to determine the association of body mass index (BMI) with 30 days and 1-year mortality outcomes of orthopedic patients after hip fracture surgery and to see if the phenomenon of obesity paradox exists in our society.

## METHODS

This is a prospective study conducted at Department of Orthopaedics, Jinnah Postgraduate Medical Centre, Karachi between Jan-2016 to Jan-2018 after the approval of ethical committee (Ref. No. F.2-81/2016-GENL/6003/JPMC, dated January 30, 2016). In this study, we included data of 490 elderly patients having age ≥60 years, who were operated for fracture of neck of femur at a tertiary care institute from Jan-2016 to Jan-2018. Patients having exceptional BMI such as less than 10 or more than 60 and polytrauma patients were excluded.

Patients were divided into different categories before doing the surgical procedure. Patients having BMI <20 Kg.m^-2^ were labelled as underweight, 20-24.99 Kg.m^-2^ normal weight, BMI 25-29.99 Kg.m^-2^ overweight, >30 obese.^18^

Data regarding ASA (American Society of Anesthesiologists) status, duration of the procedure and length of stay was also recorded. Same surgical procedure was done (bipolar hemiarthroplasty) in all patients.

Mortality at 30 days and 1-year mortality were primary study end-points. While Major adverse cardiovascular events (MACE) was secondary outcome point. Acute myocardial infarction, any cardiovascular mortality and stroke were labelled under the heading of MACE. Patients data was recorded prospectively during routine follow-up visits. If any patient was lost in follow-up, patients or his primary relative were contacted telephonically at completion of one year of surgery, if the patient is expired or he/she has changed the hospital for follow-up evaluation.

Data analysis was done using SPSS version 23. Chi-square test was used to compare qualitative variables between patient having different BMI groups. ANOVA test was used for comparison of quantitative variables between the groups. P-value ≤0.05 was taken as significant association.

## RESULTS

Out of 490 patients, there were 73 underweight patients, 146 normal weight, 154 over-weight and 117 obese patients. Mean age of patients in under-weight patients was significantly lower as compared to other BMI categories (p-value <0.0010). There was no significant difference in ASA status, and frequency of co-morbid conditions between patients within different categories (Table-I).

**Table-I:** Comparison of Baseline Variables Among Patients with Different BMI.

	Underweight (N=73)	Normal Weight (N=146)	Overweight (N=154)	Obese (N=117)	P-value
Age (Y)	62.9±9.89	66.45±8.23	65.87±6.43	67.91±7.80	<0.0001
Female Sex (%)	54 (73.9)	96 (65.7)	102 (66.2)	84 (71.8)	0.47
***ASA Status (%)***					
I-II	30 (41.1)	59 (40.4)	64 (41.6)	47 (40.1)
III	39 (53.4)	79 (54.1)	81 (52.6)	63 (53.8)
IV	4 (5.4)	08 (5.5)	9 (5.8)	7 (5.9)
***Comorbidities (%)***					
Diabetes Mellitus	18 (24.6)	40 (27.4)	37 (24.0)	33 (28.2)	0.84
Hypertension	35 (47.9)	67 (45.9)	72 (46.7)	61 (52.1)	0.76

On comparison of study outcomes, mean hospital stay was a little prolonged in underweight patients but this difference was not significant. Similarly rate of re-admission within 30 days, MACE within 30 days and 30 days’ mortality was high in underweight and lowest in obese patients. 30-day mortality rate was 2.7% in underweight, 1.3% in normal weight, 0.64% in over-weight and 0.0% in obese patients but this was not significant statistically (p-value 0.29). Mortality rate at 1-year was significantly high in under-weight patients, 34.2%, 25.9% in normal weight, 21.4% in overweight and only 14.5% in obese patients (p-value 0.009) (Table-II).

**Table-II:** Comparison of Study Outcomes Among Patients with Different BMI.

	Underweight (N=73)	Normal Weight (N=146)	Overweight (N=154)	Obese (N=117)	P-value
Hospital Stay	4.04±1.9	3.61±1.6	3.51±1.3	3.56±1.3	0.08
Re-admission within 30 days	6 (8.21)	9 (5.8)	6 (3.89)	4 (3.4)	0.39
30 days MACE	2 (2.7)	1 (0.68)	0 (0.0)	1 (0.85)	0.20
30 day Mortality	2 (2.7)	2 (1.3)	1 (0.64)	0 (0.0)	0.29
1 year Mortality	25 (34.2)	40 (25.9)	33 (21.4)	17 (14.5)	0.009

On comparison of univariate analysis, we found a significant association of age ≥65 years with 1-year mortality (odds ratio 0.40 (0.26-0.63), p-value 0.0006), male gender (odds ratio; 0.66 (0.43-1.03), p-value 0.08), and ASA status III-IV (odds ratio; 0.27 (0.16-0.45), p-value <0.0001). Regarding BMI there was significant association of BMI with 1-year mortality, in underweight patients the odds of having 1-year mortality was 3.06 (1.51-6.20) (p-value 0.002), in normal weight patient’s odds ratio was 2.22 (1.18-4.16) with p-value 0.013 by taking obesity as reference value, this association proved that obesity paradox existed in our population (Table-III).

**Table-III:** Association of Different Risk Factors with 1 Year Mortality.

	1 Year Mortality		P-value	Odds Ratio (95% CI)

	Yes	No		
Age <65 Years	69 (18.95%)	295 (81.05%)	0.0006	0.40 (0.26-0.63)
Age ≥65 Years	46 (36.50%)	80 (63.50%)		
Male Gender	44 (28.57%)	110 (71.43%)	0.08	0.66 (0.43-1.03)
Female gender	71 (21.13%)	265 (78.87)		
ASA I-II	20 (10.0%)	180 (90.0%)	<0.0001	0.27 (0.16-0.45)
ASA III-IV	95 (29.3%)	195 (70.7%)		
***BMI***				
Underweight	25 (34.2%)	48 (65.8%)	0.002	3.06 (1.51-6.20)
Normal Weight	40 (27.4%)	106 (72.6%)	0.013	2.22 (1.18-4.16)
Overweight	33 (21.4%)	121 (78.6%)	0.15	1.60 (0.84-3.05)
Obese^(Ref)^	17 (14.5%)	100 (85.5%)	----------	--------------------------

## DISCUSSION

In present cohort study, we evaluated the impact of BMI on early and 1-year mortality of patients with hip fractures. We did not find any significant association of BMI with early MACE and 30 days’ mortality. However, we found statistically significant difference in 1-year mortality rate; with highest mortality rate in underweight patients and lowest in obese patients.

A study conducted by Flodin et al. on 843 patients on one-year mortality after hip fracture surgery, reported significant mortality difference in patients with different BMI groups, with highest morality rates, 18% in normal weight patients, 16% in underweight and only 6% in normal weight patients.^19^ In their study, highest mortality was found in normal weight but in our study mortality was high in under-weight patients and after that in normal weight. Moreover, these authors included BMI <22 Kg/m^2^ in the category of underweight, while we took 20 Kg/m^2^ as cut-off to define underweight.

Another study by Prieto-Alhambra et al. reported similar results, they reported that over-weight and obese patients got the benefit of longer survival as compared to normal weight patients after hip fracture surgery.^20^ Shaparin et al. evaluated the association of BMI with complications rate after hip fracture surgery. The authors reported that BMI is a predictor of early complications after hip surgery. They found higher complications rate 23.5% in under-weight patients, 17.3% in normal weight, and only 8% in overweight patients, with little high 10% in class I obese and 14.3% in class II obese patients and 16.7% in class III obese patients. They reported U-shaped relationship between the BMI and early complications.^21^

Mouchti compared the 90 days’ mortality rate after total hip replacement surgery. The authors reported that underweight patients have 2.09 times higher risk of 90-day mortality when compared to over-weight and obese patients.^22^

Another study by Modig et al. compared the one-year morality outcomes in 17,756 patients with regard to BMI. The authors reported one year’s survival rate of 70.3% in under-weight, 77.2% in normal weight, 81.9% in over-weight and 85.2% in obese patients. The authors recommended that obesity paradox holds true for old age patients specially those having age >65 years.^23^ Other studies from U.K. and U.S. involving 409,096, and 34744 patients have concluded a positive association of obesity with 30 to 90 days’ mortality risk.^24^,^25^

Studies conducted by Belmont et al. and Meller et al. have reported higher rate of complications in super obese patients as compared to morbid obese patients.^26^,^27^ In present study we did not categorized separately super-obese patients we only labelled obese as a single category for all patients having BMI >30 Kg/m^2^, because of smaller number of obese patients in present study.

Regarding analysis of other risk factors of one-year mortality, we found significant association of age ≥65 years, ASA status III-IV with one-year mortality. However, there was weak association of gender with one-year mortality. A study by Flodin et al. also reported a significant association between advancing age with one-year mortality, while they also reported weak association between gender and mortality. Moreover, these authors reported significant association of ASA I-II with one-year mortality (odds ratio; 2.6 (1.4–5.0), p-value 0.004),^24^ our study results regarding ASA are contrary to the results of this study.

The effect of obesity paradox against adverse events has also been observed in cardiac, other surgical procedures especially in over-weight and obese population.^28^-^30^ The higher risk of mortality in under-weight patients may be explained due to the presence of any chronic illness that may be responsible for their low weight but this explanation requires further explanations. This explanation also has many objections because all patients are screened before surgery either they are medically fit for surgery or not so this explanation also does not seems correct for explaining higher mortality in these patients.^27^ Moreover there is higher prevalence of mal-nutrition in Pakistani population which might be responsible for being under-weight in our population.^31^ Further investigations are required to determine the pathophysiological causes higher mortality in under-weight patients.

### Strength of the study

The main strength of present study is the homogenous population as we only included elderly patients who presented with femur neck fractures and underwent hemiarthroplasty, while other published studies included mixed population i.e. femur neck fractures and trochanteric fractures and different procedures.

### Limitations of the study

The major limitation is that it’s a single center study with limited patients size. So there is a need to conduct a multi-centric study in Pakistan including >1000 patients to get more authenticity regarding existence of obesity paradox in orthopedic surgeries.

## CONCLUSION

BMI classification can serve as an important indicator of adverse early outcomes after hip fracture surgery. Over-weight and obese patients have better survival outcomes and have lower 1-year mortality rate.

### Author`s Contribution

**MT:** Conception of study, writing, results, interpretation bibliography, final approval of manuscript and is responsible and accountable for the accuracy or integrity of the work.

**NA:** supervision of study, validation of study

**MQAS:** review of manuscript and final approval of study.

**ARJ:** reviewed the initial and final draft and final approval of the study.
